# Model of Yield Response of Corn to Plant Population and Absorption of Solar Energy

**DOI:** 10.1371/journal.pone.0016117

**Published:** 2011-01-31

**Authors:** Allen R. Overman, Richard V. Scholtz

**Affiliations:** Agricultural & Biological Engineering Department, University of Florida, Gainesville, Florida, United States of America; Lund University, Sweden

## Abstract

Biomass yield of agronomic crops is influenced by a number of factors, including crop species, soil type, applied nutrients, water availability, and plant population. This article is focused on dependence of biomass yield (Mg ha^−1^ and g plant^−1^) on plant population (plants m^−2^). Analysis includes data from the literature for three independent studies with the warm-season annual corn (*Zea mays* L.) grown in the United States. Data are analyzed with a simple exponential mathematical model which contains two parameters, viz. *Y_m_* (Mg ha^−1^) for maximum yield at high plant population and *c* (m^2^ plant^−1^) for the population response coefficient. This analysis leads to a new parameter called characteristic plant population, *x_c_ = *1/*c* (plants m^−2^). The model is shown to describe the data rather well for the three field studies. In one study measurements were made of solar radiation at different positions in the plant canopy. The coefficient of absorption of solar energy was assumed to be the same as *c* and provided a physical basis for the exponential model. The three studies showed no definitive peak in yield with plant population, but generally exhibited asymptotic approach to maximum yield with increased plant population. Values of *x_c_* were very similar for the three field studies with the same crop species.

## Introduction

Biomass production by agronomic crops is related to a number of management factors. These factors include applied nutrients (such as N, P, and K), water availability (by rainfall or irrigation), and plant population. Many field studies have been conducted on dependence of yield on plant population for various crops. Studies with corn (*Zea mays* L.) can be found in references [1]–[6]. Additional studies have been conducted with cotton (*Gossypium hirsutum,* L.) such as [7], with the broad-leaf plant tobacco (*Nicotiana tabaccum* L.) in [8], [9], and with potato (*Solanum tuberosum* L.) in [10].

A linear-exponential model has been proposed to describe yield response of corn to plant population [11]. Data analysis was based on a field study [5], which included three plant populations. This model exhibited a peak in yield response. However, examination of data from additional studies with a greater number of plant populations, such as [1], [2], brought this assumption into question.

In this article a more suitable mathematical model is proposed that is more consistent with extensive field data. A physical basis for the model is also provided.

## Methods

The first step is to define relevant quantities: *x* is plant population, plants m^−2^; *Y* is yield of biomass (dry matter), Mg ha^−1^; and *y = Y/x* is specific yield (average yield per plant), g plant^−1^. While plant quantity is defined by the natural numbers 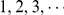, plant population (plants area^−1^) is treated as a continuous variable. The second step is to define a response function which relates the response variable *Y* to the control variable *x*. It is *assumed* that incremental change in *Y* with change in *x*, *dY/dx*, due to increase in plant population is proportional to the unfilled capacity of the system, *Y_m_ – Y*, which can be written as the first order differential equation
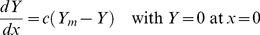
(1)where *Y_m_* is total yield capacity of the system, Mg ha^−1^; and *c* is the response coefficient, m^2^ plant^−1^. Integration of Eq. (1) leads to the *response function Y*(*x*)
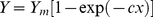
(2)with the two parameters *Y_m_* and *c*. According to this model biomass yield is bounded by 0<*Y<Y_m_*. Equation (2) can be rearranged to the linearized form
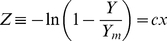
(3)


Specific yield (average yield per plant) is then defined by
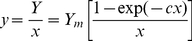
(4)which represents the *competition function y*(*x*). By Taylor series expansion of the numerator in Eq. (4) it can be shown that the intercept, *y*_o_, of *y* on *x* is given by
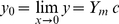
(5)


Equation (4) can now be written as
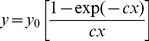
(6)


It follows that *y* is bounded by *y*_o_>*y*>0.

Equations (2) and (6) constitute the basic mathematical model that describes plant response to population. This will be used as the test of the utility of the model.

## Results

Data from three independent field studies with corn in the USA are now used to support the model. For this analysis, the Wisconsin study was chosen for the modest number of treatment populations (5 populations), and the fact that the experiment was conducted in two different locations, where the same agronomic regiment was practiced at both locations. Data from Wisconsin are used to test agreement of the model with measurements. The New York study was also singled out because of the large number of treatment populations (9 populations), and because both total and grain biomass were reported. Data from New York are used to confirm the model for both corn silage and grain. Finally, the Massachusetts study was selected, despite the relatively few treatment populations (3 populations), because of the alternate data regarding light interception through the canopy. The Massachusetts data are used to relate absorption of solar energy within the crop canopy to response of yield to plant population. Plant population is assumed uniform for each treatment and replication. Other studies, such as [4]–[10], provide similar evidence but were not selected as part of the data analysis to maintain focus and for brevity.

### Study with Corn in Northern and Southern Wisconsin

Data for this analysis are taken from studies with corn [2] in Wisconsin during the period 1994 – 1996. Experiments were conducted in the Northern zone at Spooner on Antigo silt loam (coarse-loamy over sandy or sandy-skeletal, mixed, superactive, frigid Haplic Glossudalf) and at Ashland on Manistee loamy sand (sandy over clayey, mixed, active, frigid Alfic Haplorthod); and in the Southern zone at Lancaster on Rozetta silt loam (fine-silty, mixed, superactive, mesic Typic Hapludalf) and at Arlington on Plan silt loam (fine-silty, mixed, superactive, mesic Typic Argiudoll). Two different hybrids were planted in each zone. Fertilizer applications varied among years. Yield data presented here are averages over the three years, two hybrids, and locations for each zone. Plant populations in the study were 4.45, 5.95, 7.45, 8.95, and 10.45 plants m^−2^. Biomass yields are for total plant (silage).

**Figure 1:**
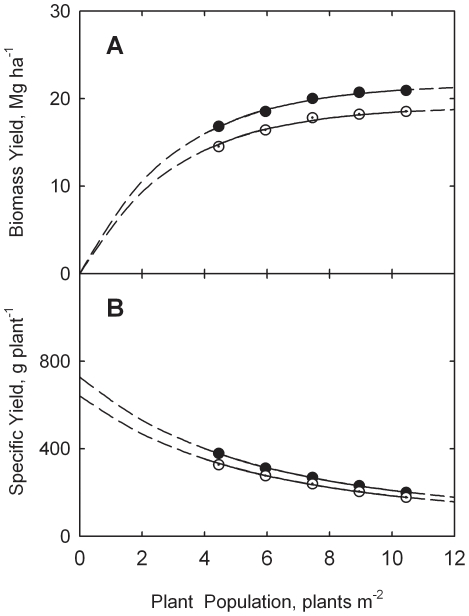
Response of biomass yield (A) and specific yield (B) to plant population for corn silage in Northern and Southern Wisconsin. Data adapted from Cusicanqui and Lauer [2]. Curves drawn from Eqs. (11) through (14).

Results are shown in Figure 1. The linearized form of the model becomes

Northern zone:
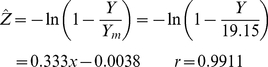
(7)


Southern zone:
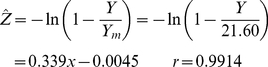
(8)where the values of *Y_m_* have been chosen to make the intercept values essentially zero to be consistent with the model. It may be noted from Eqs. (7) and (8) that the correlation coefficients are very high (*r*>0.99) and that the *c* values are essentially the same (0.333 and 0.339) for the two zones. Combination of the data for the two zones leads to

Both zones:
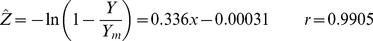
(9)


It is evident from Eqs. (7), (8) and (9) that *Y_m_* can be chosen to make the intercept arbitrarily close to 0, since the intercept values are several orders of magnitude less than the significance of the data. It appears reasonable to assume a common value of *c* = 0.336 for the two zones. Thus, it can be shown from regression theory that the optimum value of the linear parameter *Y_m_* related to a given *c* can be estimated from
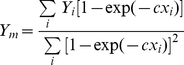
(10)which leads to 19.08 and 21.64 Mg ha^−1^ for the Northern and Southern zones, respectively. The estimation equations now become

Northern zone:
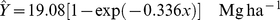
(11)

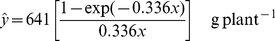
(12)


Southern zone:
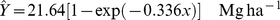
(13)

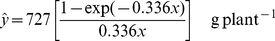
(14)


The curves in Figure 1 are drawn from Eqs. (11) through (14).

Analysis of variance can now be performed to test the hypothesis of common *c* [12]. In mode (1) individual *Y_m_* and *c* are assumed for each zone, whereas in mode (2) individual *Y_m_* and common *c* are assumed. Residual sum of squares of deviations (RSS) between measured yield (*Y_i_*) and estimated yield (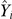) is calculated from
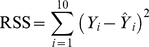
(15)


Mean sum of squares (MSS) is then defined by
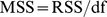
(16)where df =  degrees of freedom  =  number of observations (n) – number of parameters (p). Results are listed in Table 1. Since the variance ratio of 0.35 is considerably less the critical value F(1,6,5%) = 5.99 the hypothesis of common *c* is accepted at the 5% level. For completeness, the hypothesis of common *Y_m_* and *c* is included. The hypothesis of common *Y_m_* is rejected since the variance ratio of 161 is considerably greater than the critical value F(2,6,5%) = 5.14.

**Table 1:** Analysis of variance for the exponential model for corn in Wisconsin.

Mode	Description	p	df	RSS	MSS	F
(1)	Individual *Y_m_* and *c*	4	6	0.2533	0.04222	----
(2)	Individual *Y_m_,* Common *c*	3	7	0.2682	0.03831	----
(2) – (1)		--	1	0.0149	0.0149	0.35
(3)	Common *Y_m_* and *c*	2	8	13.83	1.729	----
(3) – (1)			2	13.58	6.79	161

The simple exponential model appears to describe the Wisconsin data rather well, as evidenced by the visual fit in Figure 1 and based on the non-linear correlation coefficients (*r*) of 0.9915 and 0.9964 for eqs. (11) and (13), respectively.

### Study with Corn at Aurora, New York

Data for this analysis are adapted from a field study with corn at Aurora, New York in 1992 and 1993 [1]. Experiments were conducted on tile-drained Honeoye silt loam (fine-loamy, mixed, mesic Glossoboric Hapludalf) with three replications of each treatment. Nine plant populations (2.96, 3.71, 4.45, 5.19, 5.93, 6.67, 7.41, 8.15, and 8.89 plants m^−2^) were included. Row spacing was 75 cm with drill spacing ranging from 15 to 45 cm. Seven hybrids (‘Funks 4385’, ‘Hytest 424’, ‘Hytest 474’, ‘Pioneer 3733’, ‘Pioneer 3592’, ‘Pioneer 3527’, and ‘Pioneer 3429’) were included, but in this analysis only average data for the seven hybrids and two years are used. Nitrogen was applied at 180 kg ha^−1^ for all plots. No irrigation was provided. Measurements were reported for silage as well as grain. All yield data are for dry matter.

**Figure 2:**
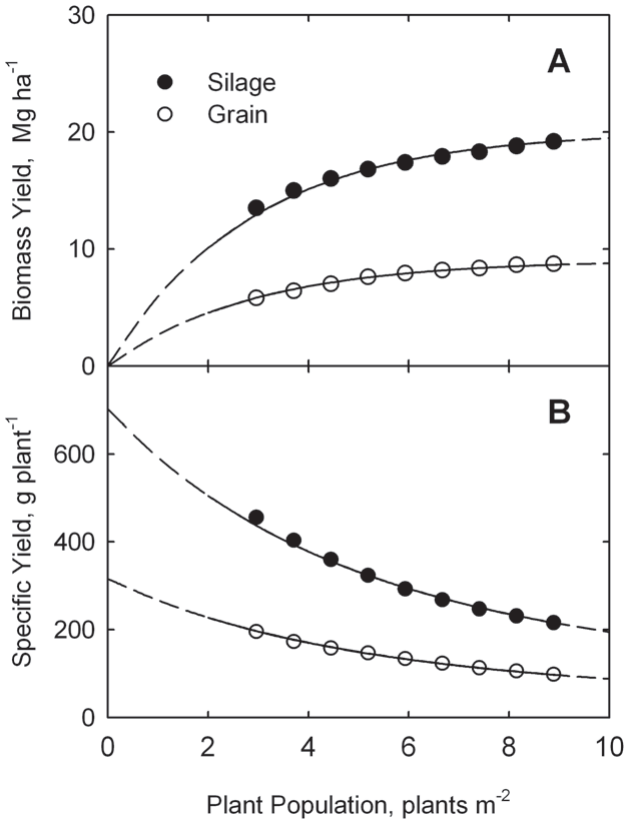
Response of biomass yield (A) and specific yield (B) to plant population for corn silage and grain at Aurora, New York. Data adapted from Cox [1]. Curves drawn from Eqs. (17) through (20).

Results are shown in Figure 2 for biomass yields and specific yields for both silage and grain. Analysis of yield data by Eq. (3) leads to an estimate of *c* = 0.350 m^2^ plant^−1^. Equation (10) is then used to estimate *Y_m_* = 20.07 Mg ha^−1^ for silage and *Y_m_* = 9.04 Mg ha^−1^ for grain. These values lead to estimation equations of

Silage:
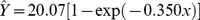
(17)

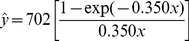
(18)


Grain:
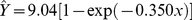
(19)

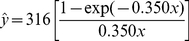
(20)


Curves in Figure 2 are drawn from Eqs. (17) through (20). The model describes the New York data rather well, as evidenced by the visual fit in Figure 2 and based on the non-linear correlation coefficients (*r*) of 0.9598 and 0.9723 for eqs. (17) and (19), respectively.

### Study with Corn at Deerfield, Massachusetts

Data for this analysis are adapted from a field study with corn grain yield at Deerfield, MA [3]. Plots were established on Hadley sandy loam (coarse-silty, mixed, nonacid, mesic Typic Udifluvent), with treatments replicated three times. ‘Agway 584S’ hybrid was planted the first week of May in 1987 and 1988. Fertilizer nitrogen of 166 kg ha^−1^ was applied each year. Plant populations were 3.0, 7.5, and 12.0 plants m^−2^. Row spacing was 75 cm. No irrigation was needed during the experiment. Measurements of photosynthetically active radiation (PAR) were made on clear days (27 August and 12 August) at ground level and at heights of 0.70, 1.20, 1.50, and 1.80 m and above the plant canopy, data for the non-shaded treatments were used in this analysis. Ear position was approximately 1.50 m above ground.

**Figure 3:**
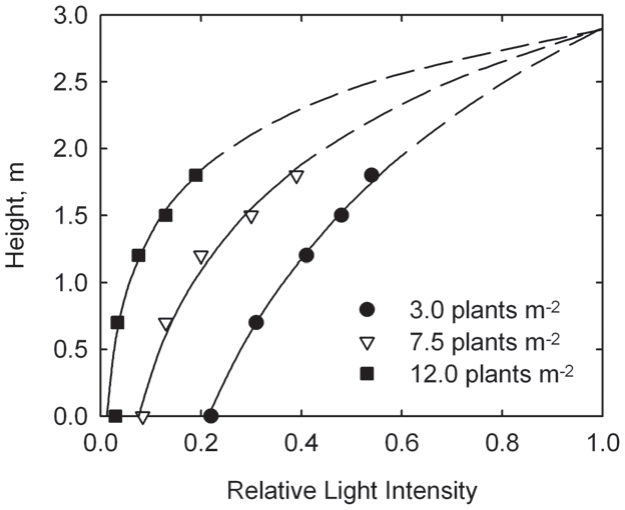
Dependence of relative light intensity on height above ground at three plant populations (*x*) at Deerfield, MA. Data adapted from Hashemi-Dezfouli and Herbert [3]. Curves drawn from Eqs. (24) through (26).

Values of relative light intensity, *f*, at various heights above ground, *Z*, are shown in Figure 3 for the three plant populations. Since relative light intensity appears to decrease somewhat exponentially with distance into the canopy, *z*, it is assumed that light intensity, I, follows 
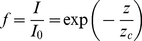
(21)where 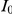 is light intensity above the canopy and z_c_ is characteristic distance into the canopy (a parameter) for the particular crop canopy. Note that *z_c_* is the position at which *f* =  exp(–1) = 0.368. Since distance into the canopy can be related to distance above ground by the simple transformation *z* = *Z_m_*–*Z*, where *Z_m_* is reference height above ground level (and above the canopy), it follows that Eq. (21) can be written as
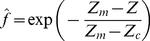
(22)where *Z_c_* is characteristic height above ground. Parameters *Z_m_* and *Z_c_* can be estimated from measurements at different heights above ground for each population. According to Eq. (22) a graph of ln *f* vs. *Z_m_ – Z* should produce a straight line.

The challenge is to estimate model parameters *Z_m_* and *Z_c_* in Eq. (22). The most rigorous procedure is nonlinear regression, from which values are listed in Table 2. Analysis of variance is now used to test the hypothesis of a common value for *Z_m_*. In this case residual sum of squares (RSS) is defined by
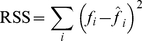
(23)where *f_i_* and 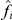 are measured and estimated values of *f*, respectively. Results are given in Table 3. Since the variance ratio 1.95 is less than the critical value F(2,9,5%) = 4.26, the hypothesis of a common *Z_m_* = 2.91 m is accepted at the 5% level. The curves in Figure 3 are drawn from

*x* = 3.0 plants m^−2^:
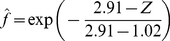
(24)
*x* = 7.5 plants m^−2^:
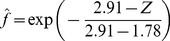
(25)
*x* = 12.0 plants m^−2^:
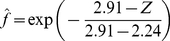
(26)


**Table 2:** Estimates of reference height (*Z_m_*) and characteristic height (*Z_c_*) at three plant populations (*x*) for absorption of solar radiation in a corn canopy at Deerfield, MA.

Mode	Description	*x*plants m^−2^	*Z_m_*m	*Z_c_*m
(1)	Individual *Y_m_* and *c*	3.0	3.00	1.01
		7.5	2.77	1.74
		12.0	2.98	2.27
(2)	Individual *Y_m_,* Common *c*	3.0	2.91	1.02
		7.5	2.91	1.78
		12.0	2.91	2.24

**Table 3:** Analysis of variance for absorption of solar radiation in a corn canopy at Deerfield, MA.

Mode	Description	p	df	RSS	MSS	F
(1)	Individual *Z_m_* and *Z_c_*	6	9	0.00118	0.000131	----
(2)	Individual *Z_c_*, Common *Z_m_*	4	11	0.00169	0.000154	----
(2) – (1)		--	2	0.00051	0.000255	1.95

The next challenge is to relate absorption of solar energy within the plant canopy to production of biomass by photosynthesis. To do this we first estimate total absorption of solar energy within the canopy by using Eqs. (24) through (26) to estimate relative intensity at ground level, 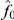. These values are summarized in Table 4. It appears that values of 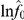 follow linear correlation with plant population. The slope of this correlation can be estimated by
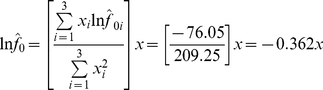
(27)where the line has been constrained to pass through the zero intercept (no plants, no absorption of solar energy). Equation (27) can be written in the equivalent form
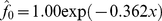
(28)


**Table 4:** Correlation of absorption of solar energy and plant yield with plant population for corn at Deerfield, MA.^1^

*x*plants m^−2^	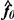	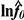	*Y*Mg ha^−1^	*y*g plant^−1^
3.0	0.214	–1.54	6.22	207
7.5	0.0761	–2.58	10.10	135
12.0	0.0130	–4.34	9.21	76.8

1 Yield data adapted from Hashemi-Dezfouli and Herbert. [3].

It is now assumed that the response coefficient in Eq. (2) is the same as the exponential coefficient in Eq. (28), viz. *c* = 0.362 for this case. Equation (10) can be used to estimate the optimum *Y_m_* for the assumed value of c using yield data from Table 4

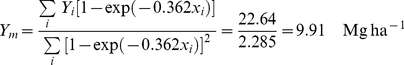
(29)


It follows that the response and competition functions are described, respectively, by
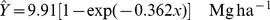
(30)

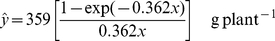
(31)


**Figure 4:**
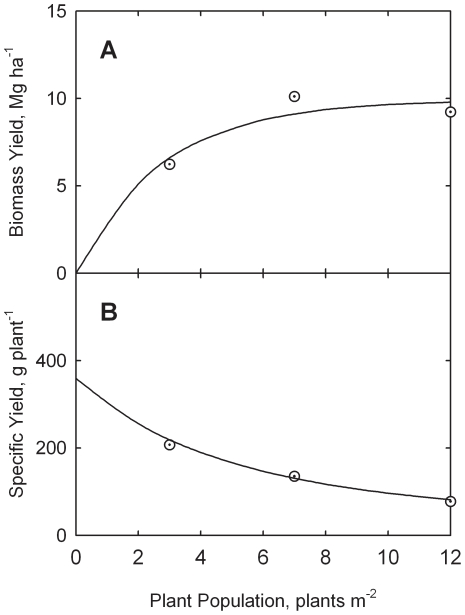
Response of biomass yield (A) and specific yield (B) to plant population for corn grain at Deerfield, MA. Data adapted from Hashemi-Dezfouli and Herbert [3]. Curves drawn from Eqs. (30) and (31).

Biomass response to plant population is shown in Figure 4, where the curves are drawn from Eqs. (30) and (31).

The model again appears consistent with the Massachusetts data, from visual inspection of Figures 3 and 4. Non-linear correlation coefficient (*r*) of 0.9271 was found for eq. (30). This alone would not be noteworthy, as there are only three plant populations; but it does indicate a strong agreement when the derived characteristic plant population (*x_c_* = 0.362) is taken in consideration, found from the analysis of the results of eqs. (24), (25) and (26) in eqs. (27) and (28).

## Discussion

Data from the northern region of the United States (Wisconsin and New York) have been used to provide an empirical basis for the simple exponential model. Data from Massachusetts were then used to provide a rational basis for the model through the absorption of solar energy within the crop canopy.

Data from a field study with corn in Wisconsin (containing five populations) have been used to test the simple exponential model of yield response to plant population. The response function shows asymptotic approach toward a maximum *Y_m_* as plant population increases. There is no evidence of a peak in biomass yield. Specific yield declines from a maximum value *y*_o_ as plant population increases, reflecting plant competition for incident solar energy. Both of these conclusions appear reasonable on intuitive grounds (see Figure 1).

Data from the study in New York (containing nine populations) lend further support for the model as applied to both corn silage and grain. Again there is no evidence of a peak in the response curves (see Figure 2).

Data from a field study with corn in Massachusetts (containing three populations) were then used to examine the relationship between absorption of solar energy within the canopy and dependence of biomass yield on plant population. Measurements showed an exponential decrease in solar intensity with position in the canopy (see Figure 3). It was possible to correlate total solar energy absorption with plant population (see Eq. (28)). It was then assumed that this exponential coefficient was the same as *c* = 0.362 plants m^−2^ for the response function for the system. This assumption appeared reasonable (see Figure 4). These results provide a physical basis for the simple exponential model.

A further characteristic of the model can now be noted. Equation (2) can be written in the equivalent form
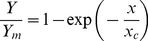
(32)where *x_c_ = *1/*c* is defined as the *characteristic plant population* of the system. It follows from the analyses that

Wisconsin:
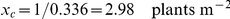



New York:
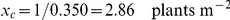



Massachusetts:
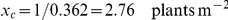



Note the close similarity between the three values for the same crop species. From the Wisconsin data, the characteristic plant population was invariant to location. Thus it is hypothesized that this property is related to the physiology of the crop, as the mechanism that would influence absorption of solar energy. It follows from Eq. (32) that biomass yield reaches 95% of maximum for *x = *3*x_c_* (*x = *8.94, 8.58 and 8.28 for the three sites respectively, at the arbitrary 95% threshold). It can also be shown that specific yield drops to 32% of maximum at *x = *3*x_c_*.

The values of linear parameters *Y_m_* and *y*_o_ should depend upon crop species, applied nutrients, and water availability. Effects of some of these factors on crop yields have been discussed elsewhere [13].

Data from other geographic regions should be used to further test the model. The model should also be tested for other plant species (such as potato, cotton, and tobacco), which is considered beyond the scope of this article. The authors plan to examine coupling of biomass yields and plant nitrogen uptake with plant population and applied nitrogen in a future publication.

## Supporting Information

Table S1 Wisconsin data for documentation of biomass yield.
(DOC)

Table S2 New York data for documentation of biomass yield.
(DOC)

Table S3 Massachusetts data for documentation of solar energy distribution.
(DOC)

Table S4 Massachusetts data for documentation of biomass yield.
(DOC)
