# Supplementary material for: Model of Yield Response of Corn to Plant Population and Absorption of Solar Energy
Source: PLoS One. 2011 Jan 31;6(1):e16117. doi: 10.1371/journal.pone.0016117 (PMC3031526; doi:10.1371/journal.pone.0016117)
Supplement: Table S1 — Wisconsin data for documentation of biomass yield. (DOC) [file pone.0016117.s001.doc]

Table S1. Wisconsin data for documentation of biomass yield.^1^

| Zone | *x*  plants m^-2^ | *Y*  Mg ha^-1^ | *y*  g plant^-1^ | $\hat{Y}$ Mg ha^-1^ | $\hat{y}$ g plant^-1^ |
| --- | --- | --- | --- | --- | --- |
| Northern | 4.45 | 14.5 | 326 | 14.80 | 332.6 |
|  | 5.95 | 16.4 | 276 | 16.50 | 277.2 |
|  | 7.45 | 17.8 | 239 | 17.52 | 235.2 |
|  | 8.95 | 18.2 | 203 | 18.14 | 202.6 |
|  | 10.45 | 18.5 | 177 | 18.51 | 177.1 |
| Southern | 4.45 | 16.8 | 378 | 16.79 | 377.3 |
|  | 5.95 | 18.5 | 311 | 18.71 | 314.4 |
|  | 7.45 | 20.0 | 268 | 19.87 | 266.7 |
|  | 8.95 | 20.7 | 231 | 20.57 | 229.8 |
|  | 10.45 | 20.9 | 200 | 20.99 | 200.9 |

^1^Data adapted from [2].

## 
