# Supplementary material for: Model of Yield Response of Corn to Plant Population and Absorption of Solar Energy
Source: PLoS One. 2011 Jan 31;6(1):e16117. doi: 10.1371/journal.pone.0016117 (PMC3031526; doi:10.1371/journal.pone.0016117)
Supplement: Table S2 — New York data for documentation of biomass yield. (DOC) [file pone.0016117.s002.doc]

Table S2. New York data for documentation of biomass yield.^1^

| Plant Component | *x*  plants m^-2^ | *Y*  Mg ha^-1^ | *y*  g plant^-1^ | $\hat{Y}$ Mg ha^-1^ | $\hat{y}$ g plant^-1^ |
| --- | --- | --- | --- | --- | --- |
| Silage | 2.96 | 13.5 | 456 | 12.95 | 437.4 |
|  | 3.71 | 15.0 | 404 | 14.59 | 393.3 |
|  | 4.45 | 16.0 | 360 | 15.84 | 356.0 |
|  | 5.19 | 16.8 | 324 | 16.81 | 323.8 |
|  | 5.93 | 17.4 | 293 | 17.55 | 296.0 |
|  | 6.67 | 17.9 | 268 | 18.13 | 271.8 |
|  | 7.41 | 18.3 | 247 | 18.57 | 250.6 |
|  | 8.15 | 18.8 | 231 | 18.91 | 232.0 |
|  | 8.89 | 19.2 | 216 | 19.18 | 215.7 |
| Grain | 2.96 | 5.80 | 196 | 5.83 | 197.0 |
|  | 3.71 | 6.41 | 173 | 6.57 | 177.2 |
|  | 4.45 | 7.02 | 158 | 7.14 | 160.4 |
|  | 5.19 | 7.61 | 147 | 7.57 | 145.9 |
|  | 5.93 | 7.93 | 134 | 7.91 | 133.3 |
|  | 6.67 | 8.19 | 123 | 8.16 | 122.4 |
|  | 7.41 | 8.35 | 113 | 8.36 | 112.9 |
|  | 8.15 | 8.64 | 106 | 8.52 | 104.5 |
|  | 8.89 | 8.72 | 98 | 8.64 | 97.2 |

^1^Data adapted from [1].
