# Supplementary material for: Model of Yield Response of Corn to Plant Population and Absorption of Solar Energy
Source: PLoS One. 2011 Jan 31;6(1):e16117. doi: 10.1371/journal.pone.0016117 (PMC3031526; doi:10.1371/journal.pone.0016117)
Supplement: Table S3 — Massachusetts data for documentation of solar energy distribution. (DOC) [file pone.0016117.s003.doc]

Table S3. Massachusetts data for documentation of solar energy distribution.^1^

| *Z*  m | *f* | | | $\hat{f}$ | | |
| --- | --- | --- | --- | --- | --- | --- |
|  | 3.0  plants m^-2^ | 7.5  plants m^-2^ | 12.0  plants m^-2^ | 3.0  plants m^-2^ | 7.5  plants m^-2^ | 12.0  plants m^-2^ |
| 0.00 | 0.22 | 0.084 | 0.030 | 0.214 | 0.076 | 0.013 |
| 0.70 | 0.31 | 0.13 | 0.034 | 0.311 | 0.141 | 0.037 |
| 1.20 | 0.41 | 0.20 | 0.076 | 0.405 | 0.220 | 0.078 |
| 1.50 | 0.48 | 0.30 | 0.13 | 0.474 | 0.287 | 0.122 |
| 1.80 | 0.54 | 0.39 | 0.19 | 0.556 | 0.374 | 0.191 |

^1^Data adapted from [3].
