# Supplementary material for: Model of Yield Response of Corn to Plant Population and Absorption of Solar Energy
Source: PLoS One. 2011 Jan 31;6(1):e16117. doi: 10.1371/journal.pone.0016117 (PMC3031526; doi:10.1371/journal.pone.0016117)
Supplement: Table S4 — Massachusetts data for documentation of biomass yield. (DOC) [file pone.0016117.s004.doc]

Table S4. Massachusetts data for documentation of biomass yield.^1^

| *x*  plants m^-2^ | *Y*  Mg ha^-1^ | *y*  g plant^-1^ | $\hat{Y}$ Mg ha^-1^ | $\hat{y}$ g plant^-1^ |
| --- | --- | --- | --- | --- |
| 3.0 | 6.22 | 207 | 6.56 | 218.8 |
| 7.5 | 10.10 | 135 | 9.25 | 123.4 |
| 12.0 | 9.21 | 77 | 9.78 | 81.5 |

^1^Data adapted from [3].
